# The Antecedents and Consequences of Metacognitive Knowledge in Mathematics Learning: A Self-Determination Perspective

**DOI:** 10.3389/fpsyg.2021.754370

**Published:** 2021-12-14

**Authors:** Yun Tang, Xiaohan Wang, Yu Fang, Jian Li

**Affiliations:** ^1^School of Psychology, Central China Normal University, Wuhan, China; ^2^Beijing Key Laboratory of Applied Experimental Psychology, Faculty of Psychology, Beijing Normal University, Beijing, China; ^3^Shenzhen Longhua Longteng School, Shenzhen, China; ^4^Yinchuan Tanglai Hui Middle School, Yinchuan, China

**Keywords:** mathematics learning, self-regulated learning (SRL), metacognitive knowledge, self-determination theory (SDT), academic performance

## Abstract

Grounded in the self-determination theory and the metacognitive and affective model of self-regulated learning, this study investigated the longitudinal relationship of self-determined motivation as the antecedent and academic performance as the consequence of metacognitive knowledge (MK) in mathematics learning. Two waves of data were collected from senior high school students (*N* = 327) in the second semester in Grades 10 and 11. A longitudinal mediation model was analyzed using structural equation modeling. Results revealed that autonomous motivation was positively related to MK of competence-enhancing strategies and negatively related to MK of avoidance strategies. Furthermore, mathematics performance was positively predicted by MK of cognitive/metacognitive strategies and negatively predicted by MK of avoidance strategies. This study expands the understanding of MK and elaborates on the dynamics between MK, self-determined motivation, and mathematics performance. Especially, this study differentiates the MK of adaptive and maladaptive strategies and examines their motivational antecedents and academic effects. Our findings also suggest that autonomous motivation has longitudinal benefits on MK.

## Introduction

Mathematics has always been highlighted for its fundamental role in many domains, such as science, engineering, and technology (Ritchie and Bates, 2013). Nevertheless, mathematics is generally viewed as challenging and mathematics underachievement in high school is common (Merenluoto and Lehtinen, 2004; Goetz et al., 2013). For senior high school students, mathematics underachievement may reduce their motivation in math learning and impact their choices of majors in higher education. Numerous efforts have been put into identifying key factors related to mathematics performance and developing approaches to enhance academic motivation and improve mathematics learning (e.g., Schoenfeld, 1992; van Velzen, 2016; Roick and Ringeisen, 2018). Empirical evidence has indicated the positive influences of metacognition on mathematics problem-solving and learning performance (Swanson, 1990; Annevirta and Vauras, 2001; Aunola et al., 2004; Neuenhaus et al., 2011; Areepattamannil and Caleon, 2013; Muncer et al., 2021). Some meta-analytical studies also have demonstrated the efficacy of metacognitive training on academic performance (e.g., Dignath and Büttner, 2008; Efklides, 2019; Theobald, 2021). Grounded in two theoretical frameworks—the metacognitive and affective model of self-regulated learning (MASRL, Efklides, 2011) and the self-determination theory (SDT, Ryan and Deci, 2017), the overarching goal of this study was to understand the longitudinal relationship between metacognition knowledge (MK), autonomous and controlled motivation, and academic performance in mathematics learning.

### MASRL Model and Metacognitive Knowledge

The conceptualization and measurement of metacognition involve multifaceted phenomena (Efklides and Vlachopoulos, 2012), including declarative knowledge about cognition (metacognitive knowledge), procedural knowledge about strategies (metacognitive skills), or feelings during the cognitive processes (metacognitive experiences). The declarative perspective of metacognition—metacognitive knowledge—is considered a core component at the person level of the MASRL model (Efklides, 2011). The metacognitive and affective model of self-regulated learning (MASRL model) emphasizes the functioning of metacognition and specifies the interactions of metacognition with cognition, motivation, and affect (Efklides, 2011). The MASRL model differentiates the person level and the task × person level of learning processes. The task × person level concerns the cognitive and metacognitive processing during specific tasks, whereas the person level consists of the more stable personal characteristics and their interactions, such as motivational beliefs, achievement goal orientation, volition, and metacognitive knowledge.

**FIGURE 1:**
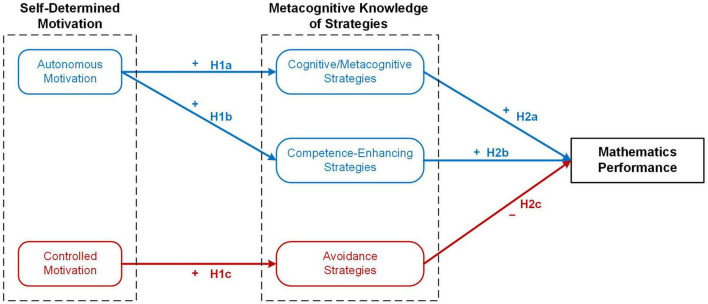
Conceptual diagram of the dual-process motivation model and the corresponding research hypotheses. Concepts and paths in blue indicate the adaptive pathway, while concepts and paths in red indicate the maladaptive pathway.

To effectively engage in the learning processes, students should possess MK on three aspects—about themselves as a learner, about demands of specific learning tasks, and about available learning strategies (Flavell, 1979; Efklides and Vlachopoulos, 2012). Among these three aspects, MK of strategies attracted the most attention of researchers as it concerns about whether students are aware of the effective strategies in achieving learning goals (Efklides, 2014). Efklides and Vlachopoulos (2012) summarized three categories of learning strategies that were usually found in mathematics learning (shown in Figure 1), namely, cognitive/metacognitive strategies, competence-enhancing strategies, and avoidance strategies. The cognitive/metacognitive strategies tap on the cognitive processes or metacognitive monitoring in problem-solving (e.g., thinking about the operations when reading the problem or evaluating the outcome after finding a solution). The competence-enhancing strategies focus on improving the overall competence in mathematics (e.g., connecting with prior knowledge or generalizing mathematical problems to everyday life). The avoidance strategies refer to the passive ways to cope with difficulties during task processing (e.g., giving up when the problem is difficult or copying the provided solutions), which represent the negative strategies that could lead to disengagement in learning (Efklides et al., 2006; Efklides and Vlachopoulos, 2012). MK, in general, or cognitive/metacognitive strategies, in particular, has been demonstrated to benefit mathematics performance (Areepattamannil and Caleon, 2013; Muncer et al., 2021; Vosniadou et al., 2021).

The academic benefits of MK may also interact with other components in self-regulated learning (SRL). Karlen (2016) examined a range of SRL components including MK, strategy use, achievement motivation, and self-efficacy. They found that students high on all SRL components achieved the highest academic performance, outperforming strategic learners (higher on MK and the frequency of strategy use) and confident learners (higher on motivation and self-efficacy), suggesting that motivational or metacognitive component alone was insufficient for superior achievement. In the next section, we review the relationship between motivation and metacognition.

### Self-Determined Motivation as an Antecedent of Metacognitive Knowledge

Self-regulated learning has traditionally emphasized the role of motivation (Zimmerman, 2000). Learners should not only know what strategies are useful and how to regulate themselves while facing difficulties but also need to have the volition to proceed with learning and manage the learning progress. Motivation is a complex construct that can be understood from several perspectives. To consider a broad conceptualization, we consulted the self-determination theory (SDT, Ryan and Deci, 2017). SDT emphasizes the quality of motivation and distinguishes between autonomous motivation and controlled motivation (Sheldon et al., 2017). In the realm of education, autonomous learners are characterized by the experience of volition and psychological freedom during learning. They engage in learning out of inherent interests (i.e., intrinsic motivation) or accept the value of learning in achieving personal goals (i.e., identified regulation). In contrast, controlled motivation is dominated by inner pressure (i.e., introjected regulation) or external authority (i.e., external regulation).

The influence factors and outcomes of autonomous motivation and controlled motivation have been widely investigated in educational contexts. Autonomous motivation can be facilitated by autonomy-supportive social context and the satisfaction of basic psychological needs, which leads to optimal consequences such as well-being, learning engagement, and better performance. In contrast, controlled motivation can be elucidated by psychologically controlled social context and the frustration of needs, while followed by non-optimal consequences such as ill-being, disengagement, and lower grades. These two pathways—the “bright” (i.e., positive or adaptive) and “dark” (i.e., negative or maladaptive) pathways—were proposed as the dual-process motivation model of SDT (Bartholomew et al., 2011; Haerens et al., 2015; Jang et al., 2016; Li et al., 2018a). According to this model, we speculated that MK of strategies may be a consequence of the motivational pathways because avoidance strategies were related to the disengagement from task processing, while the other two types of strategies were related to task engagement (Efklides and Vlachopoulos, 2012). On the one hand, learners with high controlled motivation may develop avoidance strategies that help to cope with difficulties and to avoid failure. On the other hand, autonomous learners may concentrate on self-improvement, and hence tend to enhance their cognitive/metacognitive strategies or competence-enhancing strategies. Figure 1 depicts the adaptive and maladaptive pathways between the constructs of self-determined motivation and MK of strategies.

Previous studies have supported the adaptive pathway in that the positive aspects of motivation are demonstrated to relate with MK or metacognitive strategies. For example, intrinsic motivation was found to positively associate with effort regulation, strategy use, and MK of strategies (Pintrich, 1999; Vansteenkiste et al., 2009; Kim and Pekrun, 2014; Tian et al., 2018). Some longitudinal studies found a relationship between intrinsic motivation and metacognitive strategy (Mikail et al., 2017). Motivation—in terms of task value and cost—was found to predict cognitive and metacognitive strategy use, while strategy use could not predict motivation (Berger and Karabenick, 2011). Beliefs about SRL would also predict cognitive and metacognitive strategies (Vosniadou et al., 2021). However, the effects of controlled motivation turned to be controversial in different cultures or education systems (e.g., Chirkov et al., 2003; Ryan and Deci, 2017). In Chinese secondary education, Gaokao (i.e., the national entrance examination to higher education) serves as a source of external orientation and introjected regulation that contributes to a high level of controlled motivation. Controlled motivation can be common and sometimes productive for the students (Yu et al., 2018). In the context of Chinese senior high schools, we may better observe the connection between controlled motivation and MK of adaptive and maladaptive strategies, which would expand the evidence for the dual-process motivation model of SDT.

### The Present Study

Self-regulated learning acquisition is a long-term process during which students can learn through instructions or discover as they are driven by needs or interests (Efklides, 2019). Existing longitudinal studies have concentrated mainly on the developmental changes of metacognition, but not the dynamics with the antecedents or consequences (e.g., Annevirta and Vauras, 2001; van der Stel and Veenman, 2014). Whereas cross-sectional studies have established the benefits of metacognition on mathematics performance (e.g., Muncer et al., 2021; Vosniadou et al., 2021), as well as the association between motivation and metacognition (e.g., Vansteenkiste et al., 2009; Tian et al., 2018). Exploring the longitudinal relationship between the variables would further inform the development of metacognition or facilitate the designing of training programs. This study intended to investigate the following research questions: (1) whether the self-determined motivation predicts the subsequent development of MK of strategies in mathematical learning; and (2) whether the MK of adaptive and maladaptive strategies have a diverse predictive effect on mathematics performance.

To answer the two research questions, this study employed a longitudinal design. Self-reported measures of motivation at Grade 10 and self-reported measures of MK of strategies at Grades 10 and 11 were collected from senior high school students. The mathematics exam scores throughout the year were collected as indicators of academic performance. As shown in Figure 1, two sets of hypotheses were proposed. The first set of hypotheses specified the relationship between the antecedents (i.e., autonomous and controlled motivation) and the MK of strategies according to the dual-process motivation model of SDT (e.g., Li et al., 2018a). Especially, autonomous learners would focus on learning processes and self-improvement, thus developing cognitive/metacognitive strategies and competence-enhancing strategies; whereas controlled learners may attach importance to external rewards and failures and develop avoidance strategies. The second set of hypotheses speculated the consequences (i.e., mathematics performances) of MK of strategies based on previous studies (e.g., Efklides and Vlachopoulos, 2012; Tian et al., 2018). That is, the cognitive/metacognitive strategies and competence-enhancing strategies would relate to task engagement and benefit learning, whereas the avoidance strategies would relate to disengagement and show a detrimental effect on learning. The following hypotheses were formulated.

*Hypothesis 1*: Autonomous motivation would positively predict MK of cognitive/metacognitive strategies (H1a), and positively predict MK of competence-enhancing strategies (H1b). Controlled motivation is expected to positively predict MK of avoidance strategies (H1c).

*Hypothesis 2*: MK of cognitive/metacognitive strategies would positively predict performance (H2a), while MK of competence-enhancing strategies would positively predict performance (H2b). MK of avoidance strategies is expected to negatively predict performance (H2c).

## Materials and Methods

### Participants and Procedure

Participants were students from one senior high school in a northwestern city in China. Two waves of data were collected in the spring of 2016 and 2017. The first wave was collected at the beginning of the second semester in Grade 10 (T1, *N* = 587), while the second wave was collected one year later (T2, *N* = 343). Of all participants at two time-waves, 328 students completed both measures. One participant was eliminated because the missing data rate was exceptionally high (higher than 30%), leaving a total of 327 students (202 female students, the average age at T1 was 16.35 ± 0.62 years old).

The consent of participating students and their guardians was obtained before data collection. Participants responded to questionnaires in classes with the presence of a research assistant. The research assistant gave the standardized instructions at the beginning of administration and emphasized that students had the right to stop participating at any time and the data would be held confidential. After data collection, each participant received a small gift.

### Measures

#### Metacognitive Knowledge of Strategies

The MK of strategies was measured at T1 and T2 using the Chinese adaptation of the *Metacognitive Knowledge in Mathematics Questionnaire* (MKMQ; Efklides and Vlachopoulos, 2012; Tian et al., 2018). Participants evaluated how often certain situations occurred to them in mathematics learning. Responses were made on a five-point Likert scale ranging from 1 (never) to 5 (always), with higher scores indicating a higher awareness of corresponding strategies.

The scale consisted of three subscales and 21 items. The evaluation of the psychometric properties at both time waves indicated suboptimal reliability and validity. We, therefore, revised the scale (reported in the Supplementary Material). Example items and Cronbach’s α coefficients of the revised subscales are listed in Table 1.

**TABLE 1:** Cronbach’s α and example items of measures for motivation and metacognitive knowledge (MK) of strategies.

Subscale		Example item	α	
MK of cognitive/metacognitive strategies		”When I have solved a mathematical problem, I am checking if I did the computations correctly.”	0.821	0.821
MK of competence-enhancing strategies		”When I learn something new in mathematics, I am checking how it is connected to previous lessons.”	0.702	0.743
MK of avoidance strategies		”When I do not understand what the mathematical problem requires, I give up.”	0.725	0.671
Autonomous Motivation	Intrinsic motivation to know	“For the pleasure that I experience when I read interesting authors.”	0.926	
	Intrinsic motivation to accomplish	“For the pleasure I experience while surpassing myself in my studies.”		
	Intrinsic motivation to experience stimulation	“Because I experience pleasure and satisfaction while learning new things.”		
	Identified regulation	“Because this will help me make a better choice regarding my career orientation.”		
Controlled Motivation	Introjected regulation	“To show myself that I am an intelligent person.”	0.840	
	External regulation	“Because I want to have ‘the good life’ later on.”		

#### Self-Determined Motivation

The Chinese adaptation of the *Academic Motivation Scale* (Zhang et al., 2016) was used to measure academic motivation at T1. Participants evaluated several reasons why they learned mathematics. Responses were made on a seven-point Likert scale, ranging from 1 (totally disagree) to 7 (totally agree). The original scale was comprised of seven subscales and 28 items. Because amotivation is defined as the lack of motivation and is generally regarded as different from the self-determination motivation (Vansteenkiste et al., 2009; Guay et al., 2013; Chemolli and Gagné, 2014), it was not included in this study. Example items of the subscales are listed in Table 1. We considered self-determined motivation as autonomous motivation (consisted of three intrinsic motivation subscales and identified regulation subscale) and controlled motivation (consisted of introjected regulation and external regulation subscales).

#### Mathematics Performance

To evaluate mathematics performance, a series of exam scores was obtained from the school. These exams included two monthly exams prior to T1, three monthly exams between T1 and T2, and two monthly exams after T2. Each exam was teacher-constructed, administered to all students in the same grade, and covered the instructional materials from the last monthly exam to the current exam. The contents of exams were aligned with the national curriculum for Grades 10 and 11 mathematics, spanning from set theory, algebra, to geometry. The maximum possible score was 150 on each exam.

### Data Analysis

Descriptive analysis was conducted in IBM SPSS 26.0. Measurement invariance tests and latent variable path analysis were conducted using Mplus 8.3 (Muthén and Muthén, 1998-2017). Maximum likelihood (ML) was used as the estimator and the bootstrap sample size was 5,000. The criteria for acceptable model fit were set as: CFI and TLI above 0.90, SRMR below 0.09, and RMSEA below 0.07 (Steiger, 2007; Hooper et al., 2008). We used item parcels to simplify the measurement model (Little et al., 2013; Marsh et al., 2013). The measurement model with parcels showed a good model fit [χ^2^(440) = 752.193, *p* < 0.001, CFI = 0.946, TLI = 0.935, RMSEA = 0.047, 90% *CI* = (0.041, 0.052), SRMR = 0.046].

Of the initial sample at T1, 53.95% completed the second measurement after one year at T2. The major reason for sample attrition was the class rearrangement at the beginning of Grade 11. Measurement invariance was evaluated for all measures between the students who completed both waves and who completed only T1. Strict invariance was found, suggesting that the sample attrition caused little sampling bias. The Supplementary Material presents the technical details of the data analyses.

## Results

### Preliminary Analyses

Means and standard deviations of observed variables and Pearson correlations between variables are presented in Table 2. The correlation coefficients suggested moderate correlations between motivation and MK of strategies. The exam scores exhibited small correlations with MK of strategies but no significant correlations with autonomous or controlled motivation.

**TABLE 2:** Means, SD, and correlations of study variables (*N* = 327).

	1	2	3	4	5	6	7	8	9	10	11
1. T1: Autonomous motivation											
2. T1: Controlled motivation	0.61**										
3. T1: MK of cognitive/metacognitive strategy	0.41**	0.26**									
4. T1: MK of competence-enhancing strategy	0.39**	0.20**	0.54**								
5. T1: MK of avoidance strategy	−0.26**	–0.01	−0.21**	−0.17**							
6. T2: MK of cognitive/metacognitive strategy	0.18**	0.15**	0.44**	0.28**	–0.04						
7. T2: MK of competence -enhancing strategy	0.26**	0.21**	0.33**	0.49**	–0.08	0.46**					
8. T2: MK of avoidance strategy	−0.18**	–0.01	–0.08	–0.10	0.38**	−0.16**	–0.10				
9. Exams prior to T1	0.38**	0.16**	0.25**	0.15**	−0.19**	0.15**	0.15**	−0.18**			
10. Exams between T1 and T2	0.34**	0.15**	0.23**	0.09	−0.14*	0.21**	0.16**	–0.23	0.77**		
11. Exams after T2	0.07	0.01	0.17**	0.02	–0.03	0.14**	0.05	−0.19**	0.44**	0.63**	
*M*	4.42	4.47	2.82	2.31	2.52	3.25	2.64	3.08	74.10	87.05	76.92
*SD*	1.25	1.23	0.82	0.84	0.93	0.79	0.89	0.92	22.82	18.16	21.16

*p < 0.05, **p < 0.01.

### Latent Variable Path Analysis

**FIGURE 2:**
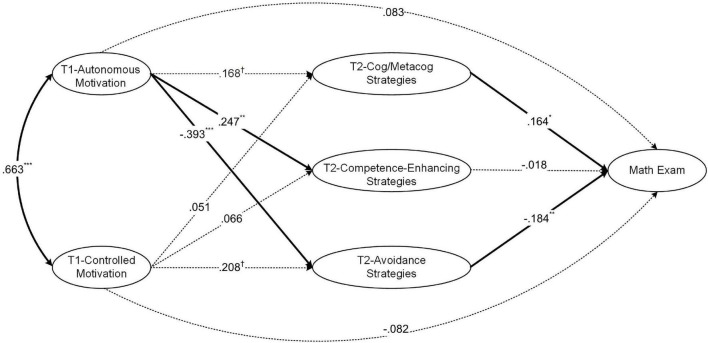
The latent variable path model between motivation, metacognitive knowledge (MK) of strategies, and mathematics performance. Standardized coefficients are presented. Solid lines indicate the significant paths and dashed lines indicate the non-significant paths. The covariate variables are not shown. T1 and T2 indicate the times of data collection. ^†^*p* < 0.10, *p < 0.05, **p < 0.01, and ***p < 0.001.

We examined the hypothetical model (Figure 1) using latent variable path analysis. A longitudinal mediation model (Cole and Maxwell, 2003) was built in which (1) motivation at T1 were considered as predictors of MK of strategies at T2, (2) MK of strategies at T2 and motivation at T1 were considered as predictors of mathematics performance after T2, and (3) the influences of the MK of strategies at T1, the exams prior to T1, and the exams between T1 and T2 were correlated with the main variables in (1) and (2). The third aspect of model setting was intended to statistically control the confounding effect of these covariate variables. Model fit was reasonable [χ^2^(447) = 825.432, *p* < 0.001; CFI = 0.935; TLI = 0.923; RMSEA = 0.051, 90% *CI* = (0.043, 0.056); SRMR = 0.078]. The model with standardized path coefficients is depicted in Figure 2.

Results indicated that autonomous motivation at T1 showed a positive longitudinal effect on MK of competence-enhancing strategies at T2 (β = 0.247, *s.e.* = 0.095, *p* = 0.010), and a negative longitudinal effect on MK of avoidance strategies at T2 (β = −0.393, *s.e.* = 0.115, *p* = 0.001). MK of cognitive/metacognitive strategies exhibited a positive relationship with mathematics performance (β = 0.164, *s.e.* = 0.080, *p* = 0.041), while MK of avoidance strategies showed a negative relationship with mathematics performance (β = −0.184, *s.e.* = 0.072, *p* = 0.011). In addition, autonomous motivation at T1 showed an indirect effect on mathematics performance through MK of strategies at T2 [total indirect effect: β = 0.096, *s.e.* = 0.039, *p* = 0.015, 95% bootstrap *CI* = (0.014, 0.191); indirect effect through MK of avoidance strategies: β = 0.072, *s.e.* = 0.033, *p* = 0.030, 95% bootstrap *CI* = (0.007, 0.149); indirect effect through the other two aspects of MK of strategies were insignificant].

## Discussion

This study aimed to investigate the longitudinal relationship between MK of strategies, self-determined motivation, and mathematics performance. Overall, we found some links between MK of competence-enhancing strategies and autonomous motivation, and between MK of avoidance strategies and autonomous motivation. Different aspects of MK of strategies further exhibited a predictive relationship with mathematics performance.

### Antecedents of Metacognitive Knowledge of Strategies

The first set of hypotheses examined whether MK of strategies may be longitudinally connected with the self-determined motivation (Hypotheses 1a–c). Results support H1b in that a small positive association was found between autonomous motivation and MK of competence-enhancing strategies. That is, for students with an autonomy in learning mathematics, their MK of competence-enhancing strategies would improve. Mixed evidence is found for H1c. Although the predictive effect of controlled motivation on MK of avoidance strategies was not statistically significant, a moderate negative association was found between autonomous motivation and MK of avoidance strategies. Students with a higher level of autonomous motivation may not easily give up in learning and attend less to maladaptive strategies, thus exhibiting a reduced level in MK of avoidance strategies. These results can be interpreted in twofold. First, our hypotheses were partially supported in that MK of competence-enhancing strategies and MK of avoidance strategies receive qualitatively different influences from autonomous motivation. Second, the findings are consistent with the “bright” pathway that autonomous motivation has an adaptive influence in academic learning (e.g., Berger and Karabenick, 2011). Especially, autonomous learners not only show a positive volition toward mathematics learning but also improve the MK of adaptive strategies and reduce the MK of maladaptive strategies.

However, H1a is not supported as the relationship between MK of cognitive/metacognitive strategies and autonomous motivation was not statistically significant. Moreover, there is no direct evidence for the “dark” pathway of controlled motivation in that controlled motivation showed no statistically significant relationship with MK of strategies. These findings may be interpreted in the context of Chinese secondary education. For senior high school students, Gaokao serves as a source of external orientation and internal controlling force that contributes to controlled motivation. Consequently, controlled motivation can sometimes become a positive antecedent of desired academic outcomes, such as academic achievement or school satisfaction (e.g., Li et al., 2018a; Yu et al., 2018). The adaptive side of controlled motivation may have counteracted its maladaptive influences, and thus showed no significant associations with MK of strategies.

### Effects on Mathematics Performance

The second set of hypotheses was about the relationship between MK of strategies and mathematics performance (Hypotheses 2a–c). Results partially supported these hypotheses in that a positive relationship was found between MK of cognitive/metacognitive strategies and performance (H2a supported) and a negative relationship was found between MK of avoidance strategies and performance (H2c supported). Whereas no significant association was found between MK of competence-enhancing strategies and performance (H2b not supported). These results expand previous findings and differentiate the effects of MK of strategies on mathematics learning. Especially, we found that MK of avoidance strategies showed a moderate and stable detrimental influence in that it predicted the decrease of mathematics performance. The negative predictive effect of MK of avoidance strategies is consistent with the belief that avoidance strategies would lead to disengagement in mathematics learning (Efklides et al., 2006; Efklides and Vlachopoulos, 2012). It prevents students to allocate effort and engage in learning when facing difficulties. In contrast, cognitive/metacognitive strategies are usually incorporated into senior high school mathematics education and show a stable relation with academic performances (Li et al., 2018b). Our results generally align with previous studies that MK of cognitive/metacognitive strategies showed a positive association with mathematics performance (e.g., Tian et al., 2018; Muncer et al., 2021).

Nevertheless, MK of competence-enhancing strategies showed no significant relationship with mathematics performance. This lack of association might imply that the mathematics performance examined in Grade 11 is not closely connected with competence-enhancing strategies. In the Chinese education system, Grade 11 is the school year approaching Gaokao in which mathematics is the required discipline for all students. Consequently, much emphasis would be placed upon excessive training rather than improving abilities in senior high schools (Yu et al., 2018). Repetitive practices and testing skills may be more influential to achieve higher exam scores. The MK of competence-enhancing strategies focuses on enhancing the general competence in mathematics, and thus showed no immediate contribution to performance.

Although we concentrated on MK of strategies and did not propose the hypotheses between motivation and mathematics performance, the model we built allowed us to examine the longitudinal effects of motivation on mathematics performance. Results suggest no direct effect of autonomous motivation or controlled motivation, but an indirect effect of autonomous motivation on mathematics performance through MK of strategies. This finding is consistent with some meta-analytical studies, which found that direct instructions of metacognitive reflection demonstrated a larger effect size than interventions focused on motivation (Dignath and Büttner, 2008; Theobald, 2021). It implies that autonomous motivation may not have a direct benefit on performance, but would take effect through other SRL components.

### Limitations and Implications

Several aspects of shortcomings should be noted when interpreting the findings. First, the sample size was limited because of the sample attrition. No difference in motivation, MK of strategies, or performance was found when checking the measurement invariance between the participants who completed both waves and who completed only T1. Nevertheless, we may have neglected other factors—such as self-efficacy or anxiety—related to the sample attrition, which potentially reduces the generalization of our results. In addition, the measures for MK of strategies showed less optimal psychometric properties. We attempted to restrain the measurement error by conducting a local revision of the scale and resorting to statistical control. Future studies should further improve the measures for MK by revising the scale or incorporating multimodal measures other than self-reports.

Second, the relationship between study variables was examined using structural equation modeling, in which the mathematics performance was assessed using teacher-constructed achievement tests and the MK of strategies and motivation were evaluated through self-report measures. While the quantitative approach is generally helpful in revealing the connections between latent constructs, other methods could also be considered to eliminate the influence of common method biases (Podsakoff et al., 2003). Essentially, MK lies at the person level of the MASRL model, while academic performances are evaluated at the task level. The relationship between MK and performance may be mediated by other SRL components at the task × person level, such as metacognitive skills, strategy use, or metacognitive monitoring and control (Efklides, 2011; van der Stel and Veenman, 2014). Future studies can extend the metacognitive processes to the task × person level and involve other metacognitive components. For example, if we require students to choose the most appropriate strategies when facing a specific learning circumstance or solving a certain type of mathematical problem (Neuenhaus et al., 2011), we could examine whether the students are aware of the optimal strategy in specific contexts. Alternatively, experiments, online assessments, think-aloud protocols, or computer log files (Veenman et al., 2014a,b) can be employed to evaluate the actual strategy used during task processing and examine whether it mediates the relationship between MK and performance.

## Data Availability Statement

The raw data supporting the conclusions of this article will be made available by the authors, without undue reservation.

## Ethics Statement

The studies involving human participants were reviewed and approved by Beijing Normal University. Written informed consent from the participants’ legal guardian/next of kin was not required to participate in this study in accordance with the national legislation and the institutional requirements.

## Author Contributions

YT: formal analysis, writing-original draft, and visualization. XW: formal analysis and writing-original draft. YF: methodology and investigation. JL: conceptualization, methodology, writing-reviewing, editing, and project administration. All authors contributed to the article and approved the submitted version.

## Conflict of Interest

The authors declare that the research was conducted in the absence of any commercial or financial relationships that could be construed as a potential conflict of interest.

## Publisher’s Note

All claims expressed in this article are solely those of the authors and do not necessarily represent those of their affiliated organizations, or those of the publisher, the editors and the reviewers. Any product that may be evaluated in this article, or claim that may be made by its manufacturer, is not guaranteed or endorsed by the publisher.
