# Supplementary material for: The Antecedents and Consequences of Metacognitive Knowledge in Mathematics Learning: A Self-Determination Perspective
Source: Front Psychol. 2021 Dec 14;12:754370. doi: 10.3389/fpsyg.2021.754370 (PMC8712685; doi:10.3389/fpsyg.2021.754370)
Supplement: Supplementary file 1 [file Table_1.PDF]

Supplementary Material

1 Revision of the Metacognitive Knowledge in Mathematics Questionnaire

The Metacognitive Knowledge (MK) of strategies was measured at T1 and T2 using the Chinese version of the Metacognitive Knowledge in Mathematics Questionnaire (MKMQ; Efklides & Vlachopoulos, 2012; Tian et al., 2018). Items on the scale asked the participants how often certain situations occurred to them in mathematics learning. Responses were made on a five-point Likert scale ranging from 1 (never) to 5 (always), with higher scores indicating a higher frequency of using corresponding strategies. The scale consists of three subscales and 21 items. Numbers of items and sample items on each scale are listed in Supplementary Table 1.

Data from all participants (N = 587) were used to evaluate the psychometric properties of MKMQ. Cronbach's α (presented in Supplementary Table 1) and CFA model fit result ($\chi^2(186) = 658.018$, $p < .001$, CFI = .835, TLI = .813, RMSEA = .067, 90%CI = (.062, .073), SRMR = .063) suggested a suboptimal reliability and validity of the scale. We inspected the factor loadings and contents of each item and removed items with a loading smaller than .50. The standardized factor loadings of each item are listed in Supplementary Table 2. The revised MKMQ has 13 items and improved psychometric properties. Cronbach's α indicated a better internal consistency within the subscales, while CFA results ($\chi^2(62) = 220.078$, $p < .001$, CFI = .922, TLI = .902, RMSEA = .067, 90%CI = (.058, .077), SRMR = .051) supported a better fit to the three-factor model.

Supplementary Table 1. Scale information and reliability estimates of MKMQ.

**Subscale**	**Original Scale**		**Revised Scale**	
	n	α	*n*	α
MK of cognitive/metacognitive strategies	10	.818	7	.821
MK of competence-enhancing strategies	5	.687	3	.702
MK of avoidance strategies	6	.706	3	.725

Note. *n* = number of items.

Supplementary Table 2. Standardized factor loadings in the original and revised MKMQ.

	Original Scale	Revised Scale
MK of cognitive/metacognitive strategies		
Item 1	.36 (.04)	N/A
Item 2	.44 (.04)	N/A
Item 3	.31 (.04)	N/A
Item 4	.66 (.03)	.68 (.03)
Item 5	.58 (.03)	.55 (.03)
Item 6	.70 (.03)	.72 (.03)
Item 7	.70 (.03)	.73 (.03)
Item 8	.58 (.03)	.56 (.03)
Item 9	.57 (.03)	.56 (.03)
Item 10	.66 (.03)	.67 (.03)

	Original Scale	Revised Scale
MK of competence-enhancing strategies		
Item 11	.65 (.03)	.67 (.03)
Item 12	.73 (.03)	.75 (.03)
Item 13	.38 (.04)	N/A
Item 14	.59 (.04)	.55 (.04)
Item 15	.40 (.04)	N/A
MK of avoidance strategies		
Item 16	0.355	N/A
Item 17	0.385	N/A
Item 18	0.746	.64 (.04)
Item 19	0.409	N/A
Item 20	0.529	.53 (.04)
Item 21	0.711	.82 (.04)

2 Sample Attrition and Measurement Invariance Check

Of the initial sample at T1, 53.95% completed the second measurement after one year at T2. MANOVA and *t*-test was performed to test the equivalence of participants who completed both waves (group 1) and who completed only T1 (group 2). Results indicated that there was no significant difference between the two groups in the motivation scores (Wilk's $\Lambda = 1.000$, $F(2, 584) = 0.104$, $p = .902$), the metacognitive knowledge scores (Wilk's $\Lambda = 1.000$, $F(3, 582) = 0.055$, $p = .983$), or the mathematics exam scores ($t(582.209)=1.538$, $p = .125$).

We further evaluated the measurement invariance of MKMQ, AMS, and mathematics performance between the two groups. Specifically, we tested models of configural invariance, metric invariance, scalar invariance, and residual invariance (Putnick & Bornstein, 2016; Vandenberg & Lance, 2000). Given the relatively large number of parameters in the measurement model and the limited sample size, we considered χ^2 test, ΔCFA, ΔRMSEA, and ΔSRMR together when evaluating the invariance hypothesis. The criteria to reject the invariance hypothesis included: a significant change in χ^2 for two nested models, and a -.01 change in CFI paired with changes in RMSEA of .015 and in SRMR of .015 (Putnick & Bornstein, 2016). Results for the invariance tests (Supplementary Table 3) supported strict measurement invariance between the two groups. Based on both results, we excluded participants who completed only T1 from the path analysis.

Supplementary Table 3. Tests of measurement invariance between participants who completed both time-waves (group 1) and participants who completed only T1 (group 2).

Model	χ^2 (*df*)	CFI	RMSEA (90% CI)	SRMR	Model comp	$\Delta\chi^2$ (Δdf)	ΔCFI	ΔRMSEA	ΔSRMR	Decision
M1: Configural Invariance	2802.51^{***} (1314)	.875	.062 (.059, .065)	.058	–	–	–	–	–	–
M2: Metric Invariance	2842.27^{***} (1353)	.875	.061 (.058, .064)	.059	M1	39.76 (39)	.000	-.001	.001	Accept
M3: Scalar Invariance	2868.50^{***} (1372)	.875	.061 (.058, .064)	.059	M2	26.23 (19)	.000	.000	.000	Accept
M4: Residual Invariance	2926.11^{***} (1411)	.873	.060 (.057, .064)	.060	M3	57.61^{*} (39)	-.002	-.001	.001	Accept

Note. $N = 587$; group 1 $n = 327$; group 2 $n = 260$.
* $p < .05$, ** $p < .01$, *** $p < .001$.

3 Longitudinal Measurement Invariance of MKMQ

Since a longitudinal mediation model was built and the MK of strategies was controlled as covariates at T1, we also examined the longitudinal measurement invariance of MKMQ between T1 and T2. Only the participants who completed both time-waves were included in this batch of measurement invariance tests. The test procedures and criteria for invariance hypothesis were the same as in the above section. Results for the invariance tests (Supplementary Table 4) supported strict measurement invariance between the two time-waves.

Supplementary Table 4. Tests of measurement invariance between T1 and T2.

Model	χ^2 (*df*)	CFI	RMSEA (90% CI)	SRMR	Model comp	$\Delta\chi^2$ (Δdf)	ΔCFI	ΔRMSEA	ΔSRMR	Decision
M1: Configural Invariance	581.65^{***} (271)	.894	.059 (.053, .066)	.065	–	–	–	–	–	–
M2: Metric Invariance	593.89^{***} (281)	.894	.058 (.052, .065)	.067	M1	12.24 (10)	-.001	-.001	.002	Accept
M3: Scalar Invariance	630.18^{***} (291)	.885	.060 (.053, .066)	.071	M2	36.29^{***} (10)	-.009	.002	.004	Accept
M4: Residual Invariance	656.90^{***} (307)	.881	.059 (.053, .065)	.079	M3	26.72 (16)	-.004	-.001	.008	Accept

Note. $N = 327$.
* $p < .05$, ** $p < .01$, *** $p < .001$.

4 Item Parceling

Item parceling technique was adopted for the Academic Motivation Scale (AMS, Zhang et al., 2016) and MKMQ. The main reason was because sample size was relatively small compared with the total number of items. It would be difficult to obtain accurate parameter estimates if all raw item scores were analyzed. In addition, we were primarily interested in the relationship between self-determined motivation and MK of strategies rather than the factor structure of each construct. Hence, we used item parcels to simplify the measurement model (Little et al., 2013; Marsh et al., 2013). The item-construct balance technique (Little et al., 2002) was employed. For AMS, four parcels each were created for autonomous and controlled motivation. For MKMQ, three parcels were created for MK of cognitive/metacognitive strategies. Since there were only three items each for MK of competence-enhancing strategies and MK of avoidance strategies, these two subscales were modelled at the item level. Supplementary Table 5 presents the results from checking the construct validity before and after parceling.

The path analysis model also included three latent variables for mathematics performance. These latent variables were evaluated by exam scores as described in the measures section. We further evaluated the full measurement model at the parcel-level and found a reasonable model fit ($\chi^2(440)=752.193$, $p < .001$, CFI = .946, TLI = .935, RMSEA = .047, 90%CI = (.041, .052), SRMR = .046). Standardized factor loadings are reported in Supplementary Table 6.

Supplementary Table 5. Item- and parcel-level CFA results for MKMQ and AMS.

Scale	Time-Wave	Level	χ^2	*df*	CFI	TLI	RMSEA	90% CI	SRMR
AMS	T1	item	1464.314^{***}	245	.844	.824	.094	(.090, .099)	.098
		parcel	88.785^{***}	19	.980	.970	.081	(.064, .098)	.032
MKMQ	T1	item	220.078^{***}	62	.922	.902	.067	(.058, .077)	.051
		parcel	43.553^{**}	24	.987	.981	.038	(.019, .056)	.038
	T2	item	230.570^{***}	62	.870	.836	.091	(.079, .104)	.074
		parcel	34.972	24	.988	.982	.037	(.000, .063)	.039

Note. * $p < .05$, ** $p < .01$, *** $p < .001$.

Supplementary Table 6. Standardized factor loadings in the full measurement model at parcel-level.

Construct/Indicator	Loading
Autonomous Motivation at T1	
Parcel 1	.88 (.01)
Parcel 2	.93 (.01)
Parcel 3	.89 (.01)
Parcel 4	.92 (.01)
Controlled Motivation at T1	
Parcel 1	.84 (.02)
Parcel 2	.85 (.02)
Parcel 3	.83 (.02)
Parcel 4	.71 (.03)
Mathematics Performance (prior to T1)	
Exam 1	.77 (.03)
Exam 2	.85 (.02)
Mathematics Performance (between T1 and T2)	
Exam 1	.74 (.03)
Exam 2	.77 (.03)
Exam 3	.78 (.03)
Mathematics Performance (after T2)	
Exam 1	.83 (.03)
Exam 2	.82 (.03)

Construct/Indicator	Loading
MK of cog/metacog strategies at T1	
Parcel 1	.77 (.03)
Parcel 2	.80 (.03)
Parcel 3	.81 (.03)
MK of comp-enhancing strategies at T1	
Item 1	.66 (.04)
Item 2	.79 (.03)
Item 3	.58 (.05)
MK of avoidance strategies at T1	
Item 1	.70 (.04)
Item 2	.54 (.05)
Item 3	.86 (.04)
MK of cog/metacog strategies at T2	
Parcel 1	.80 (.03)
Parcel 2	.79 (.03)
Parcel 3	.83 (.03)
MK of comp-enhancing strategies at T2	
Item 1	.74 (.03)
Item 2	.84 (.03)
Item 3	.54 (.05)
MK of avoidance strategies at T2	
Item 1	.83 (.04)
Item 2	.40 (.06)
Item 3	.71 (.04)

5 Metacognitive Knowledge in Mathematics Questionnaire

Please cite the following references when using this scale: (Efklides & Vlachopoulos, 2012; Tian et al., 2018).

Metacognitive Knowledge of Strategies (Cognitive/Metacognitive Strategies)

1 When I am reading a mathematical problem I am thinking whether there are various ways for solving it.
当我阅读一个数学问题时我会思考是否可用多种方法解答。

2 I pay attention to the words in the phrasing of the problem in order to figure out what is required so that I solve it (e.g., the word “and” means addition).
我会注意题目中的措辞以得到所需信息，从而作答。（如：“和”表示加法运算）

3 When I do not understand something I am asking my teacher to explain it to me so that I can go on on my own.
当我遇到不懂的地方时我会请教老师解释给我，这样我就可以继续独立作答。

4 When I have solved a mathematical problem I am checking if I did the computations correctly.

当我答完一道数学问题后我会检查是否计算正确。

5 When I find the mathematical problem complicated I am thinking the various pieces of it separately and in which sequence to put them in order to solve it.
当我遇到一道十分复杂的数学问题时，我会对它的各部分分别进行思考，依次解决它。

6 When I finish the solution of a mathematical problem I read the problem again and check if I did the operations in the order they should be done.
当我完成了一道数学问题后，我会再读一遍题来检查我是否按照题目本应该的顺序进行了计算。

7 When I finish the solution of a mathematical problem I evaluate the outcome if it is in accordance with what the problem required.
当我完成了一道数学问题，我会对结果进行检验，看是否符合题目要求。

8 When a mathematical problem is complex I am thinking in advance the operations that need to be done and in which sequence.
当一道数学问题比较复杂时，我会事先思考需要进行的运算及其顺序。

9 When I have a difficult problem to solve I am reading it many times in order to understand what the problem requires.
当我有一道难题需要解决时，我会多次阅读题目，来理解题目要求。

10 As I do computations to solve a mathematical problem I monitor myself to check whether I did them correctly so that I make corrections if needed.
当我通过计算解决一道数学问题时，我会督促自己检查正误，以便我及时纠正。

Metacognitive Knowledge of Strategies (Competence-Enhancing Strategies)

11 When I learn something new in mathematics I am checking how it is connected to previous lessons.
当我在学习新的数学知识时，我会审视它与之前课程是如何连接的。

12 When I learn something new in mathematics I am trying to compare it with other similar concepts also in mathematics (e.g., what is the difference between addition of integers and addition of decimals).
当我在学习新的数学知识时，我会尝试将它与其它相似的数学概念进行比较。（如：整数加法运算和小数加法运算的区别是什么）

13 I am playing mathematical games in magazines or in the computer.
我会在杂志或电脑上玩数学游戏。

14 When I solve mathematical problems I am thinking of other similar ones from everyday life.
当我解答数学问题时，会考虑日常生活中与之相类似的问题。

15 I like to create mathematical exercises for myself and figure out how to solve them.
我喜欢给自己出一些数学练习题，并找出解答它们的方法。

Metacognitive Knowledge of Strategies (Avoidance Strategies)

16 When I have mathematical exercises to do and I can find somewhere the solution readymade I am copying it.
当我有数学练习需要做时，我总能找到现成的答案来抄袭完成。

17 I am solving the exercises I can and I leave out the rest.
我会将我能够解答的数学问题做完，留下我解答不了的。

18 When the mathematical problem is difficult I give up.
当我碰到很难的数学问题时，我会放弃作答。

19 When I solve a mathematical problem that I do not understand I am checking how my fellow students solve it.
当我解决一个我理解不了的数学问题时，我就去看我的同学是如何作答的。

20 When I am solving a mathematical problem I do all the operations I can and then I stop even if I have not found the solution.
当我解答一道数学问题时，我在完成我能够做的所有计算后就停下来，即使还未得出答案。

21 When I do not understand what the mathematical problem requires I give up.
当我理解不了数学问题的要求时，我会放弃。

6 Academic Motivation Scale

Please cite the following references when using this scale: (Vallerand et al., 1992; Zhang et al., 2018).

Autonomous Motivation

Intrinsic Motivation to Experience Stimulation

4 Because I really like studying math
因为我真的很喜欢学数学。

11 Because for me, math is fun
因为对我来说，数学充满乐趣。

18 For the pleasure that I experience when I am taken by discussions with math teachers
因为当我跟数学老师讨论时，我沉浸其中，觉得很愉快。

25 For the "high" feeling that I experience while reading about math learning materials
因为当我读到各种有趣的数学材料时，我觉得很兴奋。

Intrinsic Motivation to Accomplish

6 For the pleasure I experience while surpassing myself in my studies
因为学习过程中的自我超越让我觉得很愉快。

13 For the pleasure that I experience while I am surpassing myself in one of my personal accomplishments
因为当我在数学的某些方面超越自己、取得成就时，我觉得很愉快。

20 For the satisfaction I feel when I am in the process of accomplishing difficult math problems
因为当我一步一步地完成那些困难的数学问题时，我觉得很满足。

27 Because high school math allows me to experience a personal satisfaction in my quest for excellence in my studies
因为高中数学能让我在追求卓越的学习过程中感到满足。

Intrinsic Motivation to Know

2 Because I experience pleasure and satisfaction while learning new things
因为学新东西时我觉得很愉快很满足。

9 For the pleasure I experience when I discover new things never seen before
因为当我发现以前从未见过的新鲜事物时，我觉得很愉快。

16 For the pleasure that I experience in broadening my knowledge about math
因为当我对数学有了更多了解时，我觉得很愉快。
23 Because my math studies allow me to continue to learn about many things that interest me
因为学习数学能够让我了解更多我感兴趣的东西。

Identified Regulation

3 Because I think that high-school math will help me better prepare for the career I have chosen
因为我认为高中数学能够让我为以后要从事的工作做好更充分的准备。
10 Because eventually it will enable me to enter the job market in a field that I like
因为学习数学最终让我能够从事自己喜欢的工作。
17 Because this will help me make a better choice regarding my career orientation
因为高中数学能帮我在决定职业方向时做出更好的选择。
24 Because I believe that high school math will improve my competence as a worker
因为我相信高中数学能提高我的工作能力。

Controlled Motivation

Introjected Regulation

7 To prove to myself that I am capable of succeeding in math
因为当我在数学上取得成功时，我能感受到自己的重要性。
14 Because of the fact that when I complete math study I feel important
为了向自己证明我有能力完成数学学习。
21 To show myself that I am an intelligent person
为了向自己证明我是个聪明的人。
28 Because I want to show myself that I can succeed in my math studies
因为我想向自己证明，我能学好数学。

External Regulation

1 Because I need at least high-school math in order to find a high-paying job later on
因为为了找到一份高薪水的工作，我至少得学习高中数学知识。
8 In order to obtain a more prestigious job later on
为了将来能找到一份社会地位更高的工作。
15 Because I want to have "the good life" later on
因为我想将来过上好日子。
22 In order to have a better salary later on
为了将来能有更高的薪水。

Amotivation

5 Honestly, I don't know; I really feel that I am wasting my time studying math
说实话，我不知道。我真的觉得我学习数学就是浪费时间。
12 I once had good reasons for studying math; however, now I wonder whether I should continue
我曾觉得学习数学有意义，但现在我开始怀疑还要不要继续学习数学。
19 I can't see why I study math and frankly, I couldn't care less
我也不知道为什么要学习数学，并且说实话，我一点都不关心。
26 I don't know; I can't understand what I am studying math
我不知道，我也不理解自己学习数学干嘛。

References

Efklides, A., & Vlachopoulos, S. P. (2012). Measurement of Metacognitive Knowledge of Self, Task, and Strategies in Mathematics. *European Journal of Psychological Assessment*, *28*(3), 227–239. https://doi.org/10.1027/1015-5759/a000145

Little, T. D., Cunningham, W. A., Shahar, G., & Widaman, K. F. (2002). To Parcel or Not to Parcel: Exploring the Question, Weighing the Merits. *Structural Equation Modeling: A Multidisciplinary Journal*, *9*(2), 151–173. https://doi.org/10.1207/S15328007SEM0902_1

Little, T. D., Rhemtulla, M., Gibson, K., & Schoemann, A. M. (2013). Why the items versus parcels controversy needn't be one. *Psychological Methods*, *18*(3), 285–300. https://doi.org/10.1037/a0033266

Marsh, H. W., Lüdtke, O., Nagengast, B., Morin, A. J. S., & von Davier, M. (2013). Why item parcels are (almost) never appropriate: Two wrongs do not make a right—Camouflaging misspecification with item parcels in CFA models. *Psychological Methods*, *18*(3), 257–284. https://doi.org/10.1037/a0032773

Putnick, D. L., & Bornstein, M. H. (2016). Measurement invariance conventions and reporting: The state of the art and future directions for psychological research. *Developmental Review*, *41*, 71–90. https://doi.org/10.1016/j.dr.2016.06.004

Tian, Y., Fang, Y., & Li, J. (2018). The Effect of Metacognitive Knowledge on Mathematics Performance in Self-Regulated Learning Framework—Multiple Mediation of Self-Efficacy and Motivation. *Frontiers in Psychology*, *9*, 2518. https://doi.org/10.3389/fpsyg.2018.02518

Vandenberg, R. J., & Lance, C. E. (2000). A Review and Synthesis of the Measurement Invariance Literature: Suggestions, Practices, and Recommendations for Organizational Research. *Organizational Research Methods*, *3*(1), 4–70. https://doi.org/10.1177/109442810031002

Vallerand, R. J., Pelletier, L. G., Blais, M. R., Brière, N. M., Senecal, C., & Vallieres, E. F. (1992). The Academic Motivation Scale: A measure of intrinsic, extrinsic, and amotivation in education. *Educational and Psychological Measurement, 52*(4), 1003–1017. https://doi.org/10.1177%2F0013164492052004025

Zhang, B., Li, Y. M., Li, J., Li, Y., & Zhang, H. (2016). The Revision and Validation of the Academic Motivation Scale in China. *Journal of Psychoeducational Assessment*, *34*(1), 15–27. https://doi.org/10.1177/0734282915575909
